# Report of a Case of Malformation of the Colon and Hand

**Published:** 1855-12

**Authors:** Wm. Walters

**Affiliations:** Frederick, Maryland


*Report of a Case of Malformation of the Colon and Hand.*
By WM. WALTERS, Frederick, Maryland.

On September 17th, 1855, I was called to Mrs. ——, then in labor, and delivered her of twin daughters between 12 and 10 o'clock A. M. The first-born, who is the subject of this report, presented the appearance of an eighth month child, very feeble, and was soon after birth almost suffocated with bloody mucus. This continued throughout the day, but late at night the bloody appearance ceased, the discharge becoming white. Her limbs and face were livid and her left wrist and hand presented a singular malformation. The metacarpal bone and phalanges of the thumb were entirely absent, and the hand was bent upwards upon the wrist at a right angle, so as to resemble very much a misplaced foot.

On the 18th she appeared stronger and better, breathing much easier. The nurse called my attention to a tumor slightly protruding from the os vaginæ, and to the fact of the bowels not having been moved, although the usual aperient had been given. I attempted to give her an enema of lard and water, but found that it flowed back without being at all tinged with meconium. I now introduced a flexible catheter into the rectum. It passed up easily about four inches, but was then arrested, and an attempt to inject through it resulted as before. Both these attempts induced strong straining and bearing down efforts, with discharge of urine and further protrusion of the tumor at the os vaginæ. The parents positively objecting to any attempts at surgical relief, and believing the case to be one of imperforate colon, I now desisted from further efforts to open the bowels, and stated to them that I thought death must soon ensue. During the day the child vomited four or five times, and made frequent straining efforts and bearing down of the perineum.

19th. Occasional vomiting, straining frequent, but no evacuation of the bowels. Had a convulsion last night.

20th. Symptoms the same.

21st. Died at 2 o'clock A. M., having lived ninety-eight hours.

*Autopsy made eight hours after death.*

The os vaginæ being entirely closed by the hymen, the apparent tumor projecting externally between the labia was found to be produced by a fluid retained in the vagina.

On opening the abdomen the uterus was found pushed up above the umbilicus, by the distended vagina; the uterus was enlarged and dilated, the greatest diameter of its cavity being half an inch, and the greatest length one and a quarter inch.

The vagina was three inches long and one inch in diameter, being thus dilated by the fluid referred to above. This was of a creamy color and consistence, and under the microscope appeared almost entirely composed of squamous mucous epithelium.

But the greatest point of interest and evident cause of death, was the anomalous conformation of the intestines. Six and a half inches below the cœcum, the colon terminated in a cul de sac. The small intestine and this portion of the colon were normal in size, direction and condition, except their peritoneal surface, which exhibited high arterial engorgement and in some places inflammation. They contained meconium, with which the small intestine was slightly distended.

The terminating point of the colon was a little below the commencement of its transverse portion, and adjacent to this point it was found again commencing in a cul de sac, and extended thence uninterruptedly seven inches to the sigmoid flexure. The whole extent of this portion was contracted almost to a cord, and was entirely empty. The rectum was also empty and contracted at its upper portion. Its length was five inches.

The surviving twin-sister is a fine healthy child, with no defect or malformation whatever.

